# Lying about money and game points by men and women and its relation to the Self-Reported Lying Scale

**DOI:** 10.3389/fpsyg.2023.1304237

**Published:** 2024-01-17

**Authors:** Eitan Elaad, Ron Kochav, Tamar Elkouby

**Affiliations:** Department of Psychology, Ariel University, Ariel, Israel

**Keywords:** lying, Ultimatum Game, money, game points, sex differences, fake fairness, Self-Reported Lying Scale, lie avoidance

## Abstract

**Introduction:**

The present study was designed to examine the effect of monetary and non-monetary endowment on lying by men and women in the Ultimatum Game. Another goal was to examine to what extent the Self-Reported Lying Scale (SRLS), described here for the first time, predicts lying in the Ultimatum Game.

**Methods:**

Examinees (162, 82 women) were allocated to four experimental conditions in a 2 × 2 factorial design. Two endowment conditions (money and game points) were crossed with two sex conditions (men and women). Participants underwent an Ultimatum Game in which they were permitted to conceal part of the endowment from an unidentified partner. Finally, participants completed the SRLS.

**Results:**

The results indicated that more cash than points were concealed from the partner, and men concealed more of their endowment than women. We further defined fake fairness in sharing that combined hiding a more significant portion of the endowment from the partner while presenting fair sharing of the remaining award. We found more fake fairness when money was shared than when points were concealed. Fake fairness is more significant for men than for women. For money and points alike, concealment was predicted by the global score of the SRLS and its five subscales (self-assessed lying ability, lie detection ability, the use of reason in lying, lie acceptability, and lie frequency).

**Discussion:**

It was suggested that a monetary endowment is more sensitive to lying than game points and involves more fake fairness. Nevertheless, the differences are quantitative, and the same response pattern exists in the two endowment conditions. Replacing money with points is a proper solution whenever a monetary endowment presents difficulties. It was further suggested that sex differences exist in lying using an asymmetric information UG, where proposers were permitted to mislead responders about their endowment. Finally, the SRLS may contribute to a better understanding of the question of who lies.

## Introduction

There is an ongoing effort to extend knowledge about the production of truth and deception and find methods to differentiate between them. As to truth production, people overestimate their ability to tell the truth convincingly (e.g., Elaad, 2019). The tendency was explained by the desire to sustain a positive self-image (Kaplar and Gordon, 2004) since the ability to be believed when telling the truth is desirable. Lie production is perceived to be more difficult than truth production, and people rate their lie-telling ability as average and sometimes even lower than average (Elaad, 2019). It was explained that the relatively low lie-telling ability is related to the belief that the inability to tell lies convincingly goes well with honesty.

Accuracy in lie detection has been found to be only slightly above the chance level, 54%, where the chance level is 50% (Bond and DePaulo, 2006). People tend to overestimate their lie-detection ability in poor lie-detection precision (e.g., Elaad, 2018a). The overestimated ability matches the general human belief that most communications are truthful and that their lack of authenticity can be revealed if they are not.

In the effort to extend the understanding of deception and deception detection, studies are often engaged with situational factors such as face-to-face interviews vs. video conferences (Dunbar et al., 2015), group differences in lying such as sex groups (e.g., DePaulo et al., 1993) or religious groups (e.g., Quinn et al., 2023), and individual variation in production and detection of deception (e.g., Köbis et al., 2019).

In this paper, we will continue linking the production of lies and self-reports of lying abilities and attitudes. Specifically, we will test the assumption that self-reported lying attributes can explain individual differences in deceptive performance. We will also clarify whether individuals scoring high on lying attributes are more inclined to use deception than lower scorers.

At the situational facet, we will investigate two situational lying effects dealing with concealing money or game points to be shared in the Ultimatum Game (UG). On the group level, we will focus on sex differences in lying. Although controversial (Canary and Hause, 1993), there is reason to believe that men are more sensitive than women to monetary gains and losses, which may unfold in the monetary UG and less so when points are shared.

On the individual level, it is broadly accepted that not many people lie frequently, and most reported not lying in the previous 24 h (Serota et al., 2010; Halevy et al., 2014; Serota and Levine, 2015; Daiku et al., 2021). Recently, Serota et al. (2023) reported a pan-cultural study (seven countries) providing evidence for a non-normal, positively skewed distribution in which most people are normatively honest, and a few prolific liars tell most lies. In addition, individual differences exist in the amount of lying people generate. In the current study, we will consider both how *many* people lie and how *much* people lie.

### Monetary and point endowment in the Ultimatum Game

The Ultimatum Game (UG) in its original form (Güth et al., 1982) provided one player (the proposer) with some monetary endowment to share with a partner (the responder). Proposers did not know the responder's identity and were free to share the money in any way they wanted. However, they were told their game partner could accept or reject their offer. The two players obtain their agreed share if the responder accepts the offer. In case of rejection, both players are left empty-handed.

The asymmetric information version of the UG (e.g., Vesely, 2014; Elaad et al., 2020) was developed to examine deception. Here, the amount of the endowment is private information for the proposer. The proposer can make any statement about the endowment to the responder. Hence, proposers may conceal part of the endowment and keep it for themselves. Then, the proposer makes an offer to the responder, who must decide whether to take or reject it. If the responder turns the offer down, both players receive nothing. It was found that, on average, proposers claim to have received a lower endowment than they indeed had.

In most asymmetric information UGs, monetary endowments were directly or indirectly involved. Jung and Vranceanu (2017) reported an example of indirect monetary endowment. Here, Experimental Currency Units were exchanged for euros at the end of the experiment. Similarly, Roth et al. (1991) compared the UG bargaining situation in four countries: The United States, Israel, Yugoslavia (Slovenia), and Japan. Such a comparison may be impaired by the different numerical scales on which payment by dinars, dollars, shekels, and yen are made. Hence, senders sharing dollars might choose different numbers than senders sharing thousands of yen or hundreds thousand dinars. The solution was to make proposals in all countries for 1,000 tokens to represent a certain sum of money. Nevertheless, the exchange of tokens for money does present difficulties. It does not consider the average standard of living in the various countries, which may have affected the endowment's psychological value differently. Dividing the endowment from its monetary incentive and replacing it with game points may be as rewarding as a small amount of cash. An attempt to use game points as endowments in the UG was reported by Elaad et al. (2020), and they found that participants deceived their playing partner to secure a game point benefit.

Kajackaite and Gneezy (2017) reported that increased endowments in cheating games do not affect lying behavior. In cheating games (e.g., Mazar et al., 2008; Fischbacher and Föllmi-Heusi, 2013), participants receive classified information and make a true or false report about this information to the experimenter. It is important to note that in cheating games, the lying option is not explicitly mentioned, and participants might be deterred from lying by the option that the experimenter has a way to detect their false report. When endowments are raised, the possible consequences increase the exposure concern (Vrij, 2008). Abeler et al. (2019) supported the idea that increased endowments do not affect lying behavior. In contrast, Kajackaite and Gneezy (2017) noted that increased endowments affect lying in deception games where lying is suggested in the game's rules, allowing the sender to send a true or false message to the receiver. The receiver must decide whether to follow the message or not.

The present study aims to elaborate on the link between endowment and deception in a situation where deception is suggested in the experimental rules and the participant does not experience exposure concerns. Specifically, we compared two types of endowment, game points and money, to understand better whether a monetary endowment (decided in a drawing) would trigger more significant lies in the UG or as cheating studies (e.g., Kajackaite and Gneezy, 2017) suggested, endowments do not affect lying behavior. The difference would likely be significant. Therefore, we may propose that group moderators such as sex might influence our monetary and game points conditions. Nevertheless, the difference in lying is quantitative, and both game points and monetary endowment mirror similar individual differences in lying. Therefore, game points may replace a small monetary endowment when the evaluation of money presents difficulties.

### Sex differences in lying

We included sex differences in lying as a variable in the present study because Dreber and Johannesson (2008) found that men lie more than women to secure a higher monetary benefit. The question is whether men lie more than women when points are at stake. The present study is designed to answer this question, but first, let us present a general review of the mixed results obtained by studying sex differences in lying.

An early meta-analysis summarizing results from widely used personality inventories between 1940 and 1992 showed that women scored slightly but consistently higher on trust than men (Feingold, 1994). Trust reflects a belief in the positive intentions of others. Burgoon et al. (2021) continued this line of reasoning, denoting that women tell more polite lies, whereas men tell frequently self-serving ones. They cited Forrest et al. (2004) to indicate that men are more successful liars than women. They explained that men tend to cover their lies with smiles more than women. Further, inauthenticity is more evident in women's smiles than in men's smiles.

Women reported telling fewer lies than men, who were more ambitious and skilled in subtle, diplomatic persuasion (Kashy and DePaulo, 1996). The bottom line is that men are less sensitive than women to honesty and, therefore, lie more. More recent meta-analyses supported this notion. A meta-analysis on honesty (Gerlach et al., 2019), which referred to 380 experiments that recorded sex differences in lying, indicated that men were 4% more deceptive than women. Another meta-analysis on sex differences in lying reported the results of 65 experimental treatments and 8,728 observations (Capraro, 2018). Capraro observed that men are significantly more likely than women to tell lies that benefit the liar at the expense of another person.

Nevertheless, the results on sex differences in lying are mixed. Elaad and Gonen-Gal (2022) found that men tended to lie more than women in one experiment but failed to replicate the result in a second experiment. Other studies failed to report sex differences in lying (e.g., Childs, 2012; Sweeney and Ceci, 2014), supporting the notion that sex differences in lying are inconsistent and negligible (Canary and Hause, 1993), and arguing that including sex differences in a study needs an apparent theoretical reason.

It is relevant to discuss sex differences in the UG. As indicated, Dreber and Johannesson (2008) reported that men are more likely than women to lie to secure a monetary gain. Eckel and Grossman (2001) found augmented generosity among women, irrespective of the partner's sex. Furthermore, a given offer is more likely to be accepted by men if it comes from a woman, interpreted as chivalry. When women are paired with women, the offer is accepted more frequently than when men offer it. The results were explained by solidarity. In a later review, Eckel and Grossman (2008) observed no significant sex differences under risk conditions. However, under low risk, women made more socially oriented decisions than men. More recently, Hasan and Ejaz (2018) strengthened the notion that women are, on average, more generous than men. Finally, Gylfason et al. (2023) found that women show a greater aversion to lying for small monetary gains than men. However, the effect disappears with increased gains.

In contrast, Saad and Gill (2001) showed that men make more generous offers to women than men, whereas women make equal offers independent of the responder's sex. A different result was presented by Solnick (2001). Solnick reported that the offers of men and women are lower for a female responder, and the highest rejection rate is found when a female proposer is paired with a female responder. Sutter et al. (2009) reported results from a bargaining experiment. No significant sex effect on behavior was reported. However, more competition and retaliation occurred when the bargaining partners were of the same sex than when they were of the opposite sex. Finally, Gylfason et al. (2013) found no sex differences in lying. The mixed results require more research on sex differences in lying. Furthermore, would the mixed results regarding the tendency to avert lying be repeated? We will also examine this question.

### Would the endowment type affect sex differences in lying?

Men's sensitivity to monetary gains and losses was demonstrated in a UG study where two players shared money (Eckel and Grossman, 2001). The results indicated that women accepted lower offers than men. In the dictator game, a version of the UG where the recipient must accept any monetary offer, men are more selfish than women (Croson and Gneezy, 2009). It was further reported that men tended to donate less than women to charity (Piper and Schnepf, 2008; De Wit and Bekkers, 2016). Other accounts have suggested that when lies benefit the liar at the expense of another person, men tend to lie more than women (e.g., Friesen and Gangadharan, 2012). Sarlo et al. (2012) examined sex differences in skin conductance and heart rate responses when engaged in UG monetary loss and gain situations. They reported that men increased skin conductance and heart rate under a loss frame, reflecting a defensive response evoked by aversive stimulation. On the other hand, a gain frame increased skin conductance associated with heart rate deceleration. These responses signal orientation, a state that primes enhanced attention and interest. Women's autonomic responses were not affected by framing.

Other studies reported that men are more sensitive than women to the excitement of gains and rewards, whereas women are more sensitive than men to the disappointment of losses and punishment (Li et al., 2007; Bobzean et al., 2014; Eneva et al., 2017; Chowdhury et al., 2019). It was found that women, relative to men, are better at delaying gratification (Byrnes et al., 1999; Silverman, 2003), which may partly explain sex differences in gains and losses.

In the UG paradigm, men are more focused on monetary endowments than women, who are more sensitive to the social context of the experiment. The different focus on gains and losses explains why men would lie more than women when the task is to secure a monetary profit.

However, would we find similar results with points as the endowment? We hypothesized that a monetary endowment would increase sex differences in lying compared to game points.

### People who refrain from lying

Finally, this paper examines the tendency to refrain from lying, regardless of the endowment condition. Lundquist et al. (2009) observed that approximately 40% to 80% of their participants lied when given a monetary incentive to lie. They concluded that some people find it psychologically challenging to lie. Serota et al. (2010) asked 1,000 people to report the number of lies they told in the last 24 h. Sixty percent reported telling no lies. Furthermore, five percent of the participants were responsible for telling almost half of all lies. It is currently agreed that refraining from lying is robust (Hurkens and Kartik, 2009), and only a few prolific liars tell most lies (Halevy et al., 2014; Serota and Levine, 2015; Daiku et al., 2021). The following hypothesis is that some participants would avoid lying, and their features would receive our attention.

### Self-Reported Lying Scale

The present study introduces the Self-Reported Lying Scale (SRLS), which combines five significant attributes of reported lying.

One attribute is the self-assessed ability to tell lies convincingly. The attribute is subjective and does not necessarily reflect everyday lie-telling behavior. Furthermore, it contrasts with the result that most lies go undetected (Bond and DePaulo, 2008). While most people tend to assign average and even low ratings to their lie-telling abilities (see Elaad, 2018a for a review), many of their lies remain unnoticed. The relatively low self-reported lie-telling ability is explained by the need to preserve a positive self-image (Kaplar and Gordon, 2004). Specifically, low ratings indicate that the rater is honest. The relatively low rating may also be explained by the belief that lying is difficult because the liar must construct a new and never-experienced story. In contrast, telling the truth is believed to be simple (Buller and Burgoon, 1996). Nevertheless, individual differences in lie-telling assessments exist, and some people assign themselves high lie-telling ratings. For example, in a recent study (Elaad, 2022), the average reported lie-telling score was above the scale midpoint of 50 (mean = 55.1, SD = 21.2). Previous results observed that self-assessed lie-telling ability correlated positively with narcissistic features (Zvi and Elaad, 2018) and negatively with religiosity (Elaad, 2018b). Furthermore, rankings of the lie-telling attribute are associated with reports of actual lying (Zvi and Elaad, 2018; Verigin et al., 2019). Specifically, people who describe themselves as good liars also reported telling more daily lies than self-reported bad liars. Furthermore, self-reported lie-telling ability predicted bigger lies to a partner in the UG (Elaad et al., 2020). Finally, it correlated positively with deliberate countermeasures in the Concealed Information polygraph Test (Elaad and Zvi, 2019). Considering sex differences in self-assessed lying abilities, men scored higher than women on the Social Adroitness scale, which was designed to pinpoint ambitious persons skilled at persuading others in a subtle diplomatic way (Kashy and DePaulo, 1996). Therefore, women, who are more sensitive than men to honesty, may undermine their lie-telling ability. The more ambitious men inflate their lie-telling ability because the skill is essential to accomplish their goals. Nevertheless, three Israeli studies with 203 men and 209 women were summarized in a meta-analysis (Elaad, 2018a). In these studies, participants were asked to rate their lie-telling ability compared to others. Answers were given on a scale ranging from 0 (*much worse than others*) to 100 (*much better than others*), with 50 (*as good as others*) as a middle score. The results show that women underestimated their lie-telling ability (weighted mean = 0.42). In contrast, men rated their lie-telling ability as good as others (weighted mean = 0.51). Sex differences in the reported lie-telling ability were significant in two studies. However, the effect size was low. In the third study, the difference was not significant.

A second attribute of reported lying is the subjective self-assessed lie-detection ability. It was observed that, on average, people tend to assess their ability to detect lies higher than the scale midpoint (see Elaad, 2018a for a review). In a recent study, almost 80% of the participants indicated they could detect lies (Fernandes et al., 2023). Fernandes et al. (2023) also asked participants to indicate their ability to detect lies on a scale ranging from 0 (unable to detect lies) to 100 (perfectly able to detect lies). The average score was close to 70%, well above the chance level. It is currently accepted that people cannot detect lies effectively at the group level and perform around chance (Bond and DePaulo, 2006, 2008). Furthermore, although exposed to deception frequently, untrained people and professionals are poor at detecting deception (Burgoon et al., 2021). People fail to detect deception because they rely on wrong cues, such as gaze aversion (Aavik et al., 2006). People also combine many, sometimes conflicting, cues into their veracity judgment (Street and Richardson, 2015). Finally, the many cues approach relies on weak cues (Verschuere et al., 2023).

Elaad and Zvi (2019) attributed inflated lie-detection estimation to the fear of being abused, threatening people's positive self-view. Specifically, people protect themselves against being deceived by believing they are good lie-catchers. It is further explained that people believe they are good lie-catchers without feedback about their lie-detection failures. Finally, the high lie detection ability bias may be associated with the so-called *truth-default* state (Levine, 2014), stating that people assume that others are honest and do not bother questioning their honesty. Wissing and Reinhard (2017) observed that psychopathy and Dark Triad personality scores were associated with higher self-assessed lie detection abilities. Kruger and Dunning (1999) suggested that overestimation is partly made by people not qualified in the field (in our case, lie detection) but need to apprehend their inability. Other results indicated that biased self-assessed lie-detection ability correlated positively with reports of frequent lying (Zvi and Elaad, 2018) and narcissism (Zvi and Elaad, 2018; Elaad et al., 2020) and negatively with religiosity (Elaad, 2018b).

To conclude, while most people inflate their assessment of their lie-detection ability, others undermine this ability. Regarding sex differences in self-assessed lie detection ability, an early meta-analysis showed that women scored slightly but consistently higher on trust scales than men (Feingold, 1994). Trust stands in line with a lower self-reported ability to disbelieve people. Nevertheless, Sweeney and Ceci (2014) reported no sex differences in lie-detection ability. In support of Sweeney and Ceci (2014), the meta-analysis of three Israeli studies (Elaad, 2018a) that asked participants to rate their lie-detection ability compared to other people showed similar ratings (weighted mean = 0.63 for both men and women). It was concluded that no sex difference exists. Men and women are biased toward enhanced lie-detection ability.

The following lying attribute incorporated in the SRLS uses rationality in lying. The four items of the present rational attribute were applied in a pilot study about sharing with an experienced interrogator in the context of the UG (Elaad, 2023, unpublished manuscript). The results indicated that the scale was rated above the scale midpoint 3 (mean = 3.46, SD = 0.74, 95% confidence interval = 3.33–3.60, *N* = 122). The results highlighted individual differences in rational processing while lying, corresponding with the broader notion of individual differences in rational processing (Cacioppo et al., 1996). More importantly, ratings of applied rationality in lying predicted enhanced lying in the UG. Finally, people would like to think of themselves as rational, with high self-reports of being rational in lying serving this belief.

Another lying attribute is lie-acceptability. McCornack and Levine (1990) observed that people differed in the extent to which they found deception acceptable. Later, Oliveira and Levine (2008) noted that lie acceptability is positively related to narcissism and negatively associated with religiosity. Recently, Quinn et al. (2023) found moderate positive correlations between lying acceptability and Machiavellianism and functional impairment at work, home, and social settings. We based our items on the statements suggested by Oliveira and Levine (2008) with some accommodations. The present scale was tested in a pilot study (Elaad, 2023, unpublished manuscript), and a low mean score was observed (mean = 2.33, SD = 0.68, 95% confidence interval = 2.18–2.48, *N* = 122). The results are explained by people's motivation to protect their belief in morality. It follows that deceiving is unacceptable. We expect the average lie acceptability score in the present study to be low.

Finally, self-assessment of frequent lying was incorporated in the SRLS, as nearly two out of every five people reported telling a lie in the last 24 h (Burgoon et al., 2021). Zvi and Elaad (2018) found that reports of frequent lying correlated positively with lie-detection ability ratings. They also reported a positive correlation between reports of frequent lying and narcissistic features. Furthermore, Gneezy et al. (2013) identified variations in individual lying over time in economic interactions. The mentioned pilot study (Elaad, 2023, unpublished manuscript) indicated that the mean score of frequent lying was lower than the scale midpoint 3 (mean = 2.09, SD = 0.70, 95% confidence interval = 1.96–2.21, *N* = 122). The low mean score may suggest that people who believe in their honesty are reluctant to report frequent lying to protect their self-image as honest people. Regarding sex differences, men reported more frequent lying than women (Kashy and DePaulo, 1996).

Although biased, it is essential to study these reported lying attributes because such reports may provide further information on how such self-perceptions influence cognition, behavior, and emotions (Bandura, 1977). Bandura's self-efficacy theory suggests that one's belief in one's ability to accomplish goals predicts success in achieving these goals. Applying this notion to perceived lying attributes suggests that people with higher scores on the five facets of the SRLS will eventually be involved in more lying than lower SRLS scorers.

The following is a summary of our hypotheses:

1. Replacing points with money would trigger more significant lies in the UG.
2. Men would lie more than women and generate bigger lies.
3. A monetary endowment would increase sex differences in lying compared to game points.
4. SRLS scores would predict lying in the UG.
5. Some participants would refrain from lying, regardless of the monetary and non-monetary conditions. The ASLS scores would distinguish between liars and non-liars.

### Design

Participants were assigned to four experimental conditions in a 2 × 2 factorial design, with two sex conditions (men and women) and two endowment conditions (money and points). The assignment to the endowment conditions was random.

## Methods

### Participants

Overall, 162 Israeli participants (82 women) participated in the study. They were equally divided into two endowment conditions (money and points). It was calculated that 75 participants would be adequate to detect a medium effect size of 0.3, with α = 0.05 and power = 0.85.

All participants were native Hebrew speakers with a mean age of 27.5 years (SD = 9.03 years). The sample consisted mainly of secular participants (115). Forty-one were traditional, and six were religious. All participants signed a consent form that promised to secure their anonymity. Participants were told they were entitled to end their participation in the study at any time without punishment. By signing the consent form, participants agreed to participate in the study.

### Self-Reported Lying Scale

The Self-Reported Lying Scale presents 20 statements answered on a 5-point sequence ranging from 1 (strongly disagree) to 5 (strongly agree), with intermediate points of 2 (disagree), 3 (no opinion), and 4 (agree). The SRLS embedded five subscales of four questions each, as follows:

1. The self-assessed lie-telling ability subscale:
  (a) *People immediately notice my lies (Reverse)*.
  (b) *My friends believe me when I lie*.
  (c) *I find it easy to convince others with my lies*.
  (d) *I lie better than most people*.

1. The self-assessed lie-detecting ability subscale presents the following:
  (a) *I am better at detecting lies than the average person*.
  (b) *People agree that I am an able lie-detector*.
  (c) *People immediately sense my inability to detect lies (Reverse)*.
  (d) *I find it easy to uncover other people's lies*.

The self-assessed lie-telling ability and the self-assessed lie-detection ability subscales were adopted from Elaad (2023). The Cronbach's α reliability reported by Elaad (2023) was 0.84 and 0.83, respectively. Using this lie-telling subscale, Elaad (2023) found that men rated their lie-telling ability higher than women.

1. Lie-acceptability items were based on Oliveira and Levine (2008) Revised Lie-Acceptability Scale:
  (a) *Lying is immoral (reverse)*.
  (b) *It is OK to lie to achieve your goals*.
  (c) *Telling the truth is always the best choice (reverse)*.
  (d) *There is nothing wrong with not telling the truth now and then*.

The scale is essential because individual differences in lie acceptability were demonstrated in adolescents (Butean et al., 2020). Furthermore, cultural differences exist in attitudes toward lying (Cantarero et al., 2018).

1. Frequent lying.
  (a) *I have no problem telling many lies*.
  (b) *I barely lie (reverse)*.
  (c) *People say I lie a lot*.
  (d) *I lie more than other people do*.
2. Being rational while lying.
  (a) *I try to be rational when I lie*.
  (b) *I hate to be rational when I am obliged to lie (reverse)*.
  (c) *My lies are thoughtful*.
  (d) *My lies make sense*.

### Procedure

The ethics committee of Ariel University approved the study. The participants were Israelis recruited from the broader community by social networks. Participants were told that the study was about sharing. After the participants agreed to participate, a female experimenter contacted the participant in a Zoom meeting and used “Google Forms” to deliver the experiment. Participants were asked to sign a consent form indicating that their identity would be kept secret and that they could withdraw from the study at any time. Participants were then presented with a brief background questionnaire (sex, age, religiosity) and were asked to complete the SRLS. Finally, half of the participants (approximately an equal number of men and women) were presented with a UG where they shared money (100 NIS, about $35 at the time of the study) with an unidentified partner. The other half shared 100 points. Note that men were informed that their partner was a man, and women shared the endowment with a woman. The Zoom meeting was performed under close surveillance of the experimenter.

The game procedure is analogous to Elaad et al.'s (2020). As such, the description of the procedure partly overlaps. Each participant received the following instructions: “In this experiment, you are matched randomly with another man (woman) called X. You will not know who X is. A sum of 100 NIS (100 points) is allocated to both of you. Only you know this is the sum of money (number of points) to be shared. X does not know the starting sum of money (number of points) and is unaware of a fair division. You should propose the sum of money (number of points) to give X, bearing in mind that the deal will be completed if only X accepts your offer. You aim to keep as much money (as many points) as possible for yourself. To this end, you may inform X that the sum of money (number of points) to be shared is < 100 NIS (100 points). If X accepts your offer, you will receive the agreed money (points). In addition, you will receive the money (points) you concealed from X. If X rejects your offer, neither of you will receive any money (points), and both will be declared losers. Remember that X cannot suggest a different money (points) division and can only accept or reject your offer. To ensure you understand the rules, please answer the following two questions before continuing:

Assume that the sum of money (number of points) you have decided to share with X is 80 NIS (points).

If you offer 30 NIS (points) to X, and X accepts your offer, you receive

_____________ NIS (points), and X receives _____________ NIS (points).

If you offer 30 NIS (points) to X, and X rejects your offer, you receive

_____________ NIS (points), and X receives_____________ NIS (points).

Now, you must decide what to offer X. Below, enter the best offer you believe will likely be accepted by X.

The number of allocated NIS (points) for sharing is _____________.

I keep ________________ NIS (points) for myself and offer X

___________ NIS (points). The final two numbers should equal the sum (number) of allocated money (points).

After completing the task, participants were thanked and debriefed.

Notes: Participants never saw any visual depiction of X.

Participants in the money condition were told that the endowment reflected actual money, and three participants winning a drawing would receive the funds to which they were entitled. Indeed, after completing the study, we performed a drawing with a random number program to decide the three winners and pay them.

## Results

Only participants who answered the two check questions correctly were included in the study. *Concealment* was defined as the difference between the initial sum of money (number of points) available for distribution (100) and the sum of money (number of points) the participant allocated for sharing with the partner. Statistics of concealment are displayed in Table 1.

**Table 1:** Means (and SDs) of the endowment (money or points) hidden from the target person in the Ultimatum Game.

	**Money**	**Points**	**Across**
Men	51.5 (28.5)	39.0 (22.1)	45.3 (28.5)
*N*	40	40	80
Women	22.7 (23.0)	10.5 (13.6)	16.6 (19.8)
*N*	41	41	82
Across	36.9 (29.5)	24.6 (23.2)	30.8 (27.2)
*N*	81	81	162

A 2 × 2 ANOVA with two between-subject factors, sex (men and women) and endowment (money and points), was conducted on the concealed sum (number of concealed points).

A significant sex effect emerged, F _(1, 158)_ = 66.3, *p* < 0.001, $\eta_{\text{p}}^{2}$ = 0.295. Table 1 shows that men concealed a larger portion of the endowment from their partners than women. Another significant main effect was obtained for endowment, F _(1, 158)_ = 12.3, *p* < 0.001, $\eta_{\text{p}}^{2}$ = 0.072. The effect indicates that more money than points was concealed from the partner. No significant interaction effect was found.

### Fair sharing

We defined a new variable, *fair sharing*. Fair sharing was computed by dividing the sum of money (number of points) the participant offered the partner by the sum of cash (number of points) they kept for themselves. The smaller the proportion, the more unfair the sharing is, as the participant retains a significant portion of the money (points). Statistics of fair sharing appear in Table 2.

**Table 2:** Means (and SDs) of fair sharing (money or points) by men and women in the Ultimatum Game.

	**Money**	**Points**	**Across**
Men	1.10 (0.66)	0.89 (0.21)	1.00 (0.50)
*N*	40	40	80
Women	0.98 (0.55)	0.81 (0.28)	0.90 (0.44)
*N*	41	41	82
Across	1.04 (0.60)	0.85 (0.25)	0.95 (0.47)
*N*	81	81	162

The lower the ratio, the less fair the sharing of the endowment.

A 2 × 2 ANOVA performed on fair sharing means, with two between-subject factors, sex (men and women) and endowment (money and points), elicited a significant endowment effect, F _(1, 158)_ = 6.95, *p* = 0.009, $\eta_{\text{p}}^{2}$ = 0.042. Inspection of Table 2 reveals that fair sharing is more conspicuous when money is involved than when points are shared. No sex differences or interaction effects were reported.

Nevertheless, fair sharing was impaired by twelve participants (7%) who offered more than half of the remaining endowment (after they allocated it for sharing) to their partners. Further examination of this group indicated that they allocated, on average, 45 percent (SD = 23.6) of their resources for sharing. On average, the remaining 150 participants allocated 71.2 (SD = 26.6) percent of their resources. We observed that concealment and fair sharing correlated positively, r _(162)_ = 0.368, *p* < 0.001, indicating that the larger the concealed endowment, the more the sharing of the remaining endowment favored the target person.

Following Ding et al. (2014), we suggested that participants who concealed a more significant portion of the endowment would display a more favorable offer of the remaining endowment to secure it from rejection. Therefore, we combined the magnitude of concealment and the fair offer into a new index, *fake fairness*. In that way, high fake fairness scores reflect a joint event of considerable concealment and a staged fair sharing of the remaining resources.

### Fake fairness

To further study fake fairness, we merged significant concealment and presentation of fair sharing into a unified measure. First, we constructed standard scales for concealment and staged fair presentation by computing standard scores relative to the respective means and standard deviations across all participants. Second, the two standard scores were added to create a combined score and ensure that each factor weighed equally in the new index. Here, high fake fairness scores reflected a more significant portion of the endowment concealed from the partner and staged fair sharing of the remaining endowment.

Table 3 presents statistics computed for the new fake fairness index.

**Table 3:** Means (and SDs) of fake fairness in sharing money and points by men and women in the Ultimatum Game.

	**Money**	**Points**	**Across**
Men	1.09 (2.05)	0.18 (1.07)	0.64 (1.69)
*N*	40	40	80
Women	−0.22 (1.61)	−1.02 (0.90)	−0.64 (1.36)
*N*	41	41	82
Across	0.43 (1.95)	−0.43 (1.16)	0.00 (1.65)
*N*	81	81	162

Higher positive scores reflect more fake fairness.

A 2 × 2 ANOVA, endowment (money and points), and sex (men and women) performed on fake fairness means revealed a significant endowment effect, F _(1, 158)_ = 13.7, *p* < 0.001, $\eta_{\text{p}}^{2}$ = 0.08. The results show more fake fairness when money is involved than when points are used. Another main effect related to sex differences, F _(1, 158)_ = 29.5, *p* < 0.001, $\eta_{\text{p}}^{2}$ = 0.157, indicated that men are more inclined than women to fake fairness. No significant interaction effect emerged. It is, therefore, suggested that men apply fake fairness when money is involved. Women will not bother to fake fairness, particularly when points are involved.

### Refraining from lying

Some participants avoided lying and allocated the entire endowment for sharing. We called this group *non-liars* (*N* = 46), contrary to *liars* (*N* = 116) who concealed part of the endowment from their partner. The number of non-liars in the money and point conditions was approximately the same, 21 (26%) and 25 (31%), respectively. However, the non-liars comprised more women (36) than men (10). Religiosity did not contribute to the number of non-liars, as none of the six religious participants were assigned to the non-liar group.

### SRLS

Another goal of the present project was to generate a lying scale to assist us with a better understanding of lying behavior. To this end, the SRLS is presented for the first time. The following was conducted to create an SRLS index: Mean and SD were computed for each participant across the 20 statements (individual total score). Then, the mean and SD of the individual total scores across participants were recorded. A similar procedure was performed for each subscale; the results are presented in Table 4. In addition, a 95% confidence interval, based on standard error units, was recorded for the total and subscale scores. Finally, the reliability of the SRLS statements was computed using Cronbach's alpha. The reliability scores are also included in Table 4.

**Table 4:** Statistics computed for the SRLS total score and five subscale scores.

	**Mean (SD)**	**95% confidence interval**	**Alpha**
Global score	2.86 (0.65)	2.76–2.96	0.90
Tell lies	3.15 (1.04)	2.99–3.31	0.85
Detect lies	3.48 (0.95)	3.33–3.63	0.86
Be rational	3.85 (0.78)	3.73–3.97	0.62
Lie acceptability	2.04 (0.76)	1.92–2.16	0.74
Frequent lying	1.82 (0.80)	1.70–1.95	0.79

*N* = 162. The confidence interval is based on standard error units.

Table 4 shows good Cronbach's alpha reliability for the global SRLS score (20 items) and four subscales (4 items each). The remaining “be rational” scale presented lower reliability than the rest.

We examined the correlations between the “be rational” scale's items to guarantee that the relatively low reliability is not due to a failing item. Results showed that all four items correlated positively and significantly. Therefore, we considered the subscale further cautiously.

For the averages, the ability to tell lies convincingly was within the midpoint range of 3 (note that the lower bound of the confidence interval is lower than three, and the upper bound is higher than 3). The results agree with earlier findings that most people assign average ratings to their lie-telling abilities (Elaad, 2018a). As expected, self-assessed lie-detection ability and being rational while lying were rated higher than the scale midpoint (the lower bound of the confidence interval is higher than 3). The results are consistent with earlier reports (Elaad, 2023, unpublished manuscript). Of no surprise were the low acceptability ratings and frequent lying (the upper bound of the confidence interval is lower than 3). The results are analogous to an earlier pilot study (Elaad, 2023, unpublished manuscript).

Next, we correlated the five subscales of the SRLS. The correlations appear in Table 5.

**Table 5:** Correlations computed for the SRLS subscale scores.

	**Tell lies**	**Detect lies**	**Be rational**	**Frequent lying**	**Lie acceptability**
Tell lies					
Detect lies	0.563^*^				
Be rational	0.519^*^	0.309^*^			
Frequent lying	0.530^*^	0.359^*^	0.318^*^		
Lie acceptability	0.438^*^	0.317^*^	0.396^*^	0.592^*^	

^*^p < 0.001, *N* = 162.

Table 5 shows that all subscales of the SRLS are positively correlated, and all correlations are significant. The results are consistent with previous findings (Elaad, 2023, unpublished manuscript).

We divided the sample between liars and non-liars and looked at differences in responding to the different SRLS scores.

Table 6 shows a consistent trend of liars scoring higher than non-liars on the SRLS. The difference is significant for two scales, being rational in lying and lie-acceptability. Nevertheless, caution is required in considering the “be rational” scale. Anyhow, results support the validity of the SRLS as an indicator for refraining from lying.

**Table 6:** Statistics computed for liars and non-liars on SRLS scores.

	**Liars mean (SD)**	**Non-liars mean (SD)**	**t _(160)_**	**Sig**	***d***
Global score	2.92 (0.64)	2.72 (0.63)	1.78	ns	0.31
Tell lies	3.19 (1.05)	3.07 (1.03)	0.67	ns	0.12
Detect lies	3.51 (0.97)	3.34 (0.97)	0.94	ns	0.17
Be rational	3.95 (0.71)	3.59 (0.87)	2.70	0.008	0.47
Lie acceptability	2.12 (0.73)	1.85 (0.79)	2.02	0.045	0.35
Frequent lying	1.83 (0.79)	1.74 (0.81)	0.63	ns	0.11

Furthermore, we examined the capability of the SRLS to predict the magnitude of concealment (lying) in the UG. To this end, we separated the money and points conditions. We applied linear regression analyses for the SRLS global score and its attributes, namely, the global SRLS score and the score of every attribute entered as the independent variable. The results appear in Table 7.

**Table 7:** Linear regression statistics describing SRLS predictions for concealing money and points in the Ultimatum Game.

	**R^2^**	**B**	**β**	**t**	**Sig**.
**Money**					
Global score	13.1%	15.82	0.36	3.44	>0.001
Tell lies	8.6%	7.89	0.29	2.72	0.008
Detect lies	8.6%	8.83	0.29	2.73	0.008
Be rational	9.9%	11.66	0.31	2.94	0.004
Lie acceptability	8.8%	11.30	0.30	2.77	0.007
Frequent lying	2.9%	6.68	0.17	1.54	0.129
**Points**					
Global score	12.1%	13.03	0.35	3.30	0.001
Tell lies	6.3%	5.91	0.25	2.31	0.023
Detect lies	1.5%	3.06	0.12	1.11	0.270
Be rational	9.0%	9.19	0.30	2.80	0.006
Lie acceptability	7.3%	8.51	0.27	2.50	0.015
Frequent lying	11.6%	9.43	0.34	3.21	0.002

Indeed, the global SRLS score predicted the magnitude of concealment of the amount of concealed money and the number of concealed points. Table 7 shows that most SRLS subscales generated significant results. Similar results were obtained from two independent samples of 81 participants, each supporting our conclusion that the SRLS attributes are capable predictors of concealment in the UG.

Finally, we examined sex differences in the self-assessed attributes of the SRLS.

The results appear in Table 8.

**Table 8:** Statistics computed for men's and women's SRLS scores.

	**Men (*N =* 80) mean (SD)**	**Women (*N =* 82) mean (SD)**	**t _(160)_**	**Sig**	***d***
Global Score	3.10 (0.64)	2.63 (0.57)	4.89	< 0.001	0.77
Tell Lies	3.50 (0.92)	2.81 (1.04)	4.47	< 0.001	0.70
Detect Lies	3.69 (0.91)	3.24 (0.97)	3.04	0.003	0.48
Be Rational	4.04 (0.71)	3.66 (0.79)	3.14	0.002	0.49
Lie Acceptability	2.23 (0.78)	1.86 (0.68)	3.25	0.001	0.51
Frequent Lying	2.03 (0.87)	1.59 (0.66)	3.56	< 0.001	0.56

*N* = 162.

Table 8 shows significant sex differences in the SRLS scores, where men scored significantly higher than women in all reported scales.

## Discussion

Lying is common. Jacobsen et al. (2018) reviewed over 100 papers on honesty and concluded that many people behave dishonestly. Nevertheless, it is a highly adaptable behavior influenced by various factors, including situational factors, group differences in lying, and individual variation in deception production.

On the situational facet, we looked for differences in lying when using money or game points. We obtained that more money than points was concealed from the target person in the UG. It is explained that money as an incentive to lie is more potent than points. Specifically, although decided by a drawing, people are more interested in winning money than points and are ready to initiate a more significant lie to accomplish their monetary win.

We defined *fair sharing* as the proportion of resources offered and kept of the remaining endowment. The smaller the proportion, the larger the portion participants kept for themselves, and sharing is less fair. We expected fair sharing in line with many studies of the UG, in which proposers make relatively fair offers.

Unexpectedly, we observed that the monetary endowment elicited more fair sharing than points. A closer inspection of the results indicated that the larger the concealed endowment, the more the remaining resources were shared in favor of the target person. We explained the result by manipulating *fake fairness*.

### Fake fairness

We observed that 12 participants (7%) offered responders more than half of the remaining endowment. This group also tended to allocate less for sharing than the other participants. A closer look at this group indicated that they consisted of eight (67%) men, and 10 (83%) were examined under the monetary condition. In two extreme cases, participants offered all the remaining endowments to the partner. However, both men allocated only 20 NIS for sharing (under the monetary condition). These two cases are examples of highly *fake fairness*. On the one hand, they were extremely eager to share the least; on the other hand, they staged extreme kindness (or fairness) in offering all the remaining endowments.

Ding et al. (2014) suggested that fear of rejection rather than concern for fairness accounted for the offering behavior. They further noted that Machiavellian personality traits played a role in that positive behavior.

Following Ding et al. (2014), we noticed that participants who concealed a more significant portion of the endowment presented a more favorable offer of the remaining endowment to the responder. We combined the magnitude of concealment and the degree of the favorable offer into a new index, *fake fairness*. In that way, high fake fairness scores reflect a joint event of considerable concealment and fair sharing of the remaining resources. We observed that more fake fairness was demonstrated under monetary incentives than points. We further noticed that men were more inclined than women to fake fairness. Men applied fake fairness mainly when money was involved. We concluded that fake fairness is employed to secure the deal and convince the partner to accept the offer.

Alternatively, the current fake fairness may be explained by feeling guilty. However, it does not make sense because the present stakes were low (game points or a small amount of money to be decided in a drawing). Second, lying was permitted by the rules of the experiment. Finally, the target person was not identified, and the proposer's anonymity was preserved.

We can find low stakes, permission to lie, and mutual anonymity online. Drouin et al. (2016) examined online deception across four different online venues (i.e., social media, online dating, anonymous chat rooms, and sexual websites). They showed that most of their participants reported not being honest on the internet, and a large majority (98%−100%) suspected that others lied online. Drouin et al. (2016) concluded that people lie “because everyone lies on the internet.”

Fake fairness is widely expected online, for example, retailers offer a deal online with a significant discount while hiding that substantial elements are not included. No doubt that fake fairness deserves more experimental attention in future research.

Finally, a monetary endowment is more sensitive to lying than game points and involves more fake fairness. Nevertheless, the differences are quantitative, not qualitative, and the same response pattern exists in the two endowment conditions. Replacing money with points is a proper solution whenever a monetary endowment presents difficulties.

### Sex differences in lying

The results show that men concealed more of the endowment from their partners than women. Men were inclined to fake fairness, whereas women were less interested in faking fairness. No sex differences were observed for fair sharing. The present study supports previous results that reported sex differences in lying using the UG. For example, in an asymmetric information UG, where proposers were permitted to mislead responders about their endowment, men were significantly more likely than women to lie to secure a monetary benefit (Dreber and Johannesson, 2008). Similarly, Jung and Vranceanu (2017) reported more lies offered by men than by women.

Men's fake fairness manipulation is explained by men's added sensitivity and increased focus on gains and rewards, a notion supported by other studies (Li et al., 2007; Bobzean et al., 2014; Eneva et al., 2017; Chowdhury et al., 2019). It was suggested that men are more excited than women by monetary gains related to their lower ability to delay gratification (Byrnes et al., 1999; Silverman, 2003). To secure their concealed endowment, men more than women used fake fairness, believing the manipulation would convince the partner to accept their offer.

### Refraining from lying

It was not surprising that some participants found it psychologically difficult to lie even when permitted to do so. We observed that irrespective of the endowment condition, 46 (28%) of our participants allocated the entire endowment (100 NIS or 100 points) to share with their partner. However, refraining from lying was more noteworthy among women (36 of 82) than among men (10 of 80). Somewhat unexpected was the finding that none of the six religious participants refrained from lying.

Our results match other reports about individual differences in lying. Lundquist et al. (2009) reported that some people find it psychologically costly to lie under various conditions. Serota et al. (2010) asked participants to report the number of lies they told in the last 24 h. Sixty percent reported telling no lies. Refraining from lying is a robust phenomenon (Hurkens and Kartik, 2009), and only a few prolific liars tell the most lies (Halevy et al., 2014; Serota and Levine, 2015; Daiku et al., 2021).

### SRLS

Correlating SRLS subjective attributes with actual low-stakes deceptive behavior is infrequent, and the present study was designed to contribute in this respect. The contribution entails devising the SRLS presented here for the first time.

The global SRLS score is reliable across all 20 items. It provided impressive predictions of the money and points concealed from the partner in the UG. Specifically, high SRLS scorers tended to conceal more money than low SRLS scorers in the monetary condition and more points in the game points condition. The independence of the two samples highlights the results. It was observed that men tended to score higher than women on the SRLS global score, which may explain why men concealed more from their partners than women. People who scored high on the various facets of the SRLS may feel confident applying their perceived abilities and attitudes and behave accordingly, irrespective of the endowment (monetary or non-monetary). The significant sex difference in the SRLS scores may also explain the manipulative behavior we call fake fairness. The results indicated that men were more inclined than women to fake fairness.

The self-assessed lie-telling ability was rated similarly to the scale midpoint, indicating that people need more confidence to deliver lies convincingly. The results support previous findings (e.g., Elaad, 2018a) despite the accepted notion that most lies go undetected (Bond and DePaulo, 2008). The relatively low assessment of lie-telling ability was explained by the belief that lying is challenging, whereas truth-telling is simple (Buller and Burgoon, 1996). Another explanation for the low-rated lie-telling ability is based on the illusion of transparency (Gilovich et al., 1998). The illusion refers to the sender's feelings of being unable to prevent the detection of their lies and the overestimation of the receiver's ability to detect their lies. Finally, people tend to believe they are honest, and by assessing their lie-telling ability as low, they preserve their honest self-image. The lie-telling ability estimates predicted the production of lies in the present UG study. Specifically, participants who scored high on the self-assessed lie-telling ability tended to conceal more money and more points from their partners. Finally, there were sex differences in the self-assessed lie-telling ability, where men scored higher than women. Similar results were obtained in two earlier studies but not in a third study (Elaad, 2018a). The higher confidence of men in their lie-telling ability helps to explain the manipulative behavior of men to secure their offer against rejection (fake fairness).

The inflated lie detection ability was demonstrated here once again. In previous accounts, it was found that objectively, people perform only slightly better than chance when trying to detect others' lies (Bond and DePaulo, 2006, 2008). However, subjectively, they report being able to detect lies (Elaad, 2003, 2009). In a recent study, Fernandes et al. (2023) explained the bias by the *truth-default* state (Clare and Levine, 2019), meaning that people assume that others are honest most of the time and tend to trust the sender. It follows that most lies remain undetected, and in the absence of feedback on the successful lying of others, responders believe they are able detectors of lies. Another explanation for the inflated estimation is the fear of being easily deceived, which threatens people's positive self-view (Elaad and Zvi, 2019). Regarding the predicted performance in the game, lie-detection ability estimates predicted concealing money but not concealing points. Finally, men scored higher than women on the lie-detection scale, contrasting the results of three earlier studies that reported no difference (Elaad, 2018a).

Being rational in lying was another attribute incorporated in the SRLS. The attribute originated from the broader notion of individual differences in rational processing (Cacioppo et al., 1996). The four scale items generated an average above the middle score, suggesting that responders are biased toward considering themselves rational. The scale's reliability was lower than the other scales; however, an additional analysis of the scale items' intercorrelation indicated that all four items correlated positively and significantly. Finally, it predicted the concealed endowment in two independent conditions: money and points.

Another lying attribute is lie-acceptability (McCornack and Levine, 1990). Lie acceptability is an attitude toward lying that varies from entirely unacceptable to a most lenient view of deception (Butean et al., 2020). It is, therefore, expected that some people would consider lying to be more acceptable than others. The lie-acceptability scale correlated positively with narcissism and negatively with religiosity (Oliveira and Levine, 2008). It is, therefore, justified to include the scale in the SRLS. The lie-acceptability scale was based on statements suggested by Oliveira and Levine (2008) with some changes. In the present study, acceptability was rated low, which agrees with people's self-view as moral persons and their motivation to sustain it. Individual differences in lie-acceptability predicted lying in two independent endowment conditions: money and points.

In addition, there were sex differences in lie-acceptability, where men scored higher than women. The results agree with men's higher fake fairness scores than women's. Nevertheless, Oliveira and Levine (2008) did not identify sex differences in lie-acceptability.

In the present study, we used a scale that asked participants to report how frequently they lie and incorporated it into the SRLS. As expected, participants rated their lying behavior low, agreeing with an honest self-view. Individual differences in frequent lying predicted the extent of concealed points but failed to predict the amount of concealed money. People who admit frequent lying feel free to conceal game points when the experimenter permits lying. The situation becomes complicated regarding money because lying about money may surpass the boundaries of honesty.

It is clear from the present results and earlier studies that the self-assessed abilities and attitudes are biased and do not necessarily reflect the actual state of the responder. However, studying these biased beliefs is essential because they may predict behavior. In the present study, the global SRLS scores and most subscale scores predicted behavior under two independent money and point endowment conditions, thus supporting the validity of the SRLS in predicting behavior. It is necessary to continue this line of research in different lying situations other than UG, for example, cheating studies, different types of lies, different incentive levels, and different groups of participants (other than Hebrew-speaking Israelis). Such a research program will enhance our knowledge about the boundaries in which the SRLS can be applied.

Finally, the present correlational design cannot answer the question of causality. Specifically, are the predictions of lying by the SRLS scores unaffected, or are participants committed to their stated SRLS attitudes and later accommodated their behavior accordingly? It remains for future research to answer the question of causality. Meanwhile, we may enlist Bandura's self-efficacy theory (Bandura, 1977) in favor of the argument that lying behavior is not a direct result of SRLS scores but a more complex feature. According to Bandura, people's belief in their ability to achieve goals facilitates success in achieving these goals.

### Limitations

The present study has limitations. One prominent limitation is its language. The SRLS was presented in Hebrew to Israeli Hebrew-speaking participants. The English version presented in Appendix A1 is a translation that tried to be as accurate as possible. Nevertheless, it should be tested on English-speaking participants and translated into other languages to determine whether it can be widely applied.

The literature suggests that age influences the tendency to deceive (e.g., Chen et al., 2023). Nevertheless, age and deception were not correlated, r _(162)_ = 0.05, *p* > 0.05.

Other factors, such as personality traits, may predict deception. Unfortunately, the present study did not examine personality traits and should be included in future research.

The study was conducted over Zoom meetings. Comparing deception in different modalities suggests that the modality changes people's behavior (i.e., Burgoon et al., 2002; Dunbar et al., 2015). For example, Dunbar et al. (2015) found that face-to-face credibility assessments by professionals in a cheating game were more accurate than video conference interviews. Therefore, it is suggested that the present study be reexamined under different modalities, particularly in face-to-face interactions.

## Data availability statement

The original contributions presented in the study are included in the article/supplementary material, further inquiries can be directed to the corresponding author.

## Ethics statement

The studies involving humans were approved by Ariel University Ethics Committee. The studies were conducted in accordance with the local legislation and institutional requirements. The participants provided their written informed consent to participate in this study.

## Author contributions

EE: Conceptualization, Data curation, Formal analysis, Investigation, Methodology, Project administration, Software, Supervision, Validation, Writing—original draft, Writing—review & editing, Resources, Visualization. RK: Conceptualization, Data curation, Investigation, Project administration, Supervision, Validation, Writing—review & editing. TE: Conceptualization, Data curation, Investigation, Project administration, Supervision, Validation, Writing—review & editing.

## Appendix

**Appendix A1:** In this questionnaire you are asked to state your position toward lying (Please indicate to what extent you agree with what is said in each statement. Do this by marking the answer that best reflects your position.).

	**Strongly disagree**	**Disagree**	**No opinion**	**Agree**	**Strongly agree**
	**1**	**2**	**3**	**4**	**5**
(1) I try to be rational when I lie	1	2	3	4	5
(2) I have no problem telling many lies	1	2	3	4	5
(3) People immediately notice my lies (R)	1	2	3	4	5
(4) I am better at detecting lies than the average person	1	2	3	4	5
(5) Telling the truth is always the best choice (R)	1	2	3	4	5
(6) I barely lie (R)	1	2	3	4	5
(7) I hate to be rational when I am obliged to lie (R)	1	2	3	4	5
(8) Lying is immoral (R)	1	2	3	4	5
(9) My friends believe me when I lie	1	2	3	4	5
(10) People agree that I am an able lie- detector	1	2	3	4	5
(11) I find it easy to convince others with my lies	1	2	3	4	5
(12) People say I lie a lot	1	2	3	4	5
(13) My lies are thoughtful	1	2	3	4	5
(14) It is OK to lie to achieve your goals	1	2	3	4	5
(15) People immediately sense my inability to detect lies (R)	1	2	3	4	5
(16) I lie better than most people	1	2	3	4	5
(17) There is nothing wrong with not telling the truth now and then	1	2	3	4	5
(18) I lie more than other people do	1	2	3	4	5
(19) My lies make sense	1	2	3	4	5
(20) I find it easy to uncover other people's lies	1	2	3	4	5

## Scope statement

The present study examined the effect of money and points on sex differences in lying to an unidentified partner. Results indicated that men lied more than women and that more cash than points was concealed. The effects of money and points on lying are systematically compared for the first time. The results demonstrate that whenever money presents difficulties, points may replace money. Furthermore, earlier results on sex differences in lying are mixed. The present results may add to the notion that such differences exist. We further defined fake fairness in sharing that combined hiding a larger portion of the endowment from the partner while presenting fair sharing of the remaining award. We found more fake fairness when money was shared than when points were concealed. Fake fairness is more significant for men than for women. For money and points alike, concealment was predicted by the global score of the Self-Reported Lying Scale (SRLS) and by most of its five subscales (self-assessed lying ability, self-assessed lie detection ability, being rational in lying, lie acceptability, and lie frequency). The SRLS is described here for the first time, and it may contribute to a better understanding of the question of who lies.
